# Editorial: Scientific integrity in nutritional research

**DOI:** 10.3389/fnut.2023.1323265

**Published:** 2023-11-10

**Authors:** Sergej M. Ostojic, Nina Cecilie Øverby

**Affiliations:** Department of Nutrition and Public Health, Faculty of Health and Sports Sciences, University of Agder, Kristiansand, Vest-Agder, Norway

**Keywords:** nutritional epidemiology, food policy, nutritional methodology, public health, precision nutrition

Scientific Integrity in Nutritional Research is a *Frontiers in Nutrition* Research Topic aimed to address various issues related to the robustness of nutritional science, from methodological issues with field-specific research and counseling, to managing conflict of interest and problems with scientific decision making in public health nutrition. Our Research Topic gathered 30 authors from North America, Europe and Asia that provided a mix of original research, review and perspective papers that timely suit co-editors invitation, exploring topics in nutritional epidemiology, food policy and economics, and nutritional methodology. Addressing the aforementioned topics holds the potential to fortify evidence-based knowledge within the realm of human nutrition as a scientific field, and perhaps pave the way for the establishment of elevated scientific standards and a heightened sense of public responsibility, echoing the sentiments expressed by Ioannidis (1).

An original paper by a Chinese research group (Lu et al.) delved into the international research landscape of the past decade concerning vitamin D and its implications on reproductive health. The researchers meticulously analyzed over 1,800 articles and reviews, revealing a significant diversity in terms of represented subdisciplines, authors' backgrounds, research frontiers, and the quality of peer-reviewed journals where these papers were disseminated. While the field of study has garnered substantial global attention, it has displayed an uneven progression, marked by multidisciplinary research hotspots primarily focusing on risk factors and detrimental consequences of vitamin D deficiency. Nevertheless, the report emphasizes the need for more robust investigations. Specifically, it suggests delving into the heterogeneity of vitamin D deficiency across populations, enhancing the utility of vitamin D-related biomarkers, and adopting a mechanistic approach to scrutinize the effects of vitamin D on health indicators. This significant report reiterates the call for the design of more comprehensive cause-and-effect studies within the realm of nutritional sciences. Furthermore, it underscores the importance of embracing personalized approaches, aligning with previous views of Bassaganya-Riera et al. (2).

Neumann et al. conducted an extensive review focusing on the significance of placebo, nocebo, and psychosocial context effects in the realms of medicine and nutritional research. The study sheds light on the psycho-neurological underpinnings of these effects, intricately influenced by variables such as the nature of dietary modifications, environmental settings, and patient surroundings. The findings underline the crucial relevance of these factors in outcome research and nutritional guidance, particularly in situations where the implementation of randomized control trials or appropriate control groups is unfeasible. The authors strongly advocate for the integration of considerations pertaining to placebo, nocebo, and psychosocial context effects into the education and daily practices of nutrition counseling. This becomes especially pertinent in investigations concerning the unique counseling requirements of individuals.

Another review article within this Research Topic look into the systematic review process of the U.S. Department of Agriculture's Nutrition Evidence Systematic Review Branch (NESR), exemplifying the utilization of a diverse range of expertise and experiences while effectively managing potential conflicts of interest (Obbagy et al.). Through collaborative efforts involving federal and public stakeholders, as well as expert groups, and the meticulous execution of systematic reviews, NESR has emerged as a pivotal initiative in informing evidence-based decisions by the U.S. government concerning public health nutrition. This encompasses pivotal contributions to the development of the *Dietary Guidelines for Americans*. The article also underscores the imperative need for other organizations to contemplate adopting a similar approach, perhaps modeled after NESR, to ensure a collaborative process that adeptly addresses potential or perceived conflicts of interest among all stakeholders involved.

A thought-provoking perspective article by Temple critically assesses diverse research methods in nutritional science, thoroughly scrutinizing the advantages and limitations of the mechanistic approach in elucidating various diet-health interactions. The author provides several illustrative examples where the mechanistic approach has demonstrated limited efficacy as a tool for elucidating effective dietary interventions for health protection. Noteworthy instances include the relationships between dietary fats and obesity, saturated fat and coronary heart disease, Mediterranean diet and cardiovascular disease, type 2 diabetes and dietary fiber, as well as cancer and micronutrients. The article intriguingly contrasts the potential shortcomings of the mechanistic approach with the strengths of observational studies. This contrast suggests that a synergistic utilization of observational studies (such as prospective cohort studies) and randomized-controlled trials may offer a more favorable strategy for generating pertinent insights into the intricate interplay between nutrition and health.

Finally, a research paper by Ko et al. investigated the reliability and validity of the Korean version of the Children's Eating Behavior Questionnaire (K-CEBQ) for children with anorexia. This parent-reported measure is designed to assess variations in eating style, and the researchers conducted two rounds of the study, surveying over 350 participants online. They demonstrated good internal consistency reliability and temporal stability of the K-CEBQ, indicating its reliability for assessing the eating behavior of children with this eating disorder. The paper also calculated the optimal cut-off values for the two category scores of the K-CEBQ, food approach and food avoidance. This provides a practical tool for healthcare professionals that could aid in diagnosing children with anorexia and assessing their treatment progress. The article emphasizes the need for future studies to consider other confounding factors (besides age and gender) that may influence anorexia features in children.

The current Research Topic offers a comprehensive overview of advancements in the realm of scientific integrity within nutritional research, advocating for a robust and practical approach from both clinical and public health standpoints. By adopting optimal strategies for research data generation and analysis, while also embracing a personalized approach such as precision nutrition, and considering diverse diet-specific neuro-psychological factors, the field can be revitalized. This rejuvenation holds the potential to bolster public policy, shed light on the broader population, and ultimately contribute to a more informed and healthier society.

## Author contributions

SO: Writing—original draft. NØ: Writing—review & editing.

## References

[1] Ioannidis JPA. The challenge of reforming nutritional epidemiologic research. JAMA. (2018) 320:969–70. 10.1001/jama.2018.11025 30422271

[2] Bassaganya-Riera J Berry EM Blaak EE Burlingame B Le Coutre J van Eden W. Goals in nutrition science 2020-2025. Front Nutr. (2021) 7:606378. 10.3389/fnut.2020.606378 33665201 PMC7923694

